# Epidemiology and Cytokine Levels among Children with Nosocomial Multidrug-Resistant *Acinetobacter baumannii* Complex in a Tertiary Hospital of Eastern China

**DOI:** 10.1371/journal.pone.0161690

**Published:** 2016-08-31

**Authors:** Chao Fang, Xuejun Chen, Mingming Zhou

**Affiliations:** Clinical Laboratory Department, Children's Hospital of Zhejiang University School of Medicine, Hangzhou, Zhejiang Province, China; University of Malaya, MALAYSIA

## Abstract

**Background and aim:**

The present study was aimed at assessing the characteristics of children with nosocomial multidrug-resistant *Acinetobacter baumannii* complex (MDR ABC) in a tertiary hospital of eastern China. MDR ABC poses a serious threat to public health. However, information on nosocomial MDR ABC in children is lacking.

**Method:**

This study retrospectively reviewed the cases in a tertiary hospital of eastern China between January 1, 2011, and December 31, 2014 (excluding outpatients).

**Results:**

A total of 377 non-duplicated nosocomial ABC isolates were collected from various samples including 200 (53.1%) MDR ABC isolates. Moreover, 158 of the 200 MDR ABC isolates were collected from intensive care units (ICUs; MDR constituent ratios, 62.5%), while 98 of the 200 MDR ABC isolates were collected from children older than 1 year (MDR constituent ratios, 62.8%). Multivariate logistic analysis revealed that being in the surgical intensive care unit (SICU), prolonged hospital stay, surgical intervention, and mechanical ventilation were independent risk factors for MDR acquisition among children with nosocomial ABC. The interleukin (IL)-6 level of children with nosocomial MDR ABC was significantly lower than that of the children with nosocomial non-MDR ABC.

**Conclusion:**

Nosocomial MDR ABC infection is a serious concern in pediatric patients. Being in the SICU, prolonged hospital stay, surgical intervention, and mechanical ventilation increased the risk of nosocomial MDR ABC. IL-6 might be involved in developing nosocomial MDR ABC among children.

## Introduction

*Acinetobacter baumannii* complex (ABC) is a nonfermenting Gram-negative aerobic coccobacillus that has great potential for nosocomial spread. Multidrug-resistant (MDR) ABC is a rapidly emerging pathogen in hospitals with antimicrobial drugs used in therapeutics, particularly in intensive care units (ICUs) [1–4]. ABC is frequently observed as a nosocomial infection that is associated with high mortality and hospitalization cost [5, 6]. According to a prospective study, the 30-day mortality of infections associated with non-MDR *A*. *baumannii* and MDR *A*. *baumannii* was 30% and 50%, respectively [1]. The rise in nosocomial MDR ABC infections has become a major clinical concern worldwide.

Although previous studies have demonstrated drug-resistance acquisition in ABC and its outcomes [7–12], only limited data focus on children with MDR ABC. Additionally, Th1/Th2 cytokines play an important role in anti-infection immunity, and Th1/Th2 balance theory has been widely accepted in academia and confirmed [13]. Based on a prospective study, interleukin (IL)-2, IL-6, and IL-10 are effective biomarkers to rule out sepsis and intracranial infection [14]. So, changes in serum Th1/Th2 cytokine profiles and levels were expected in children with MDR ABC infection. In this study, we retrospectively reviewed the cases in a tertiary hospital of eastern China between January 1, 2011, and December 31, 2014 (excluding outpatients) and evaluated the epidemiological features, risk factors, and serum Th1/Th2 cytokine levels among children with nosocomial MDR ABC. This study is the first specialized report on the characteristics that might be used to prioritize infection control practices and select reasonable empirical therapies.

## Material and Methods

### Study design

The study was conducted in a 1000-bed tertiary hospital in eastern China between January 1, 2011, and December 31, 2014. All cultures obtained from inpatients that yielded ABC were considered as nosocomial infection as identified from medical and laboratory records. Also relevant information (including patient’s age (<18 years), gender, specimen type, wards, and major risk factors) was gathered from medical and laboratory records. All patients with nosocomial ABC positive clinical cultures were identified retrospectively based on the first positive culture. To accurately assess the cytokine levels, patients with cancer and autoimmune disease, which could interfere with the level of cytokines, were excluded [15]. At the onset of nosocomial ABC infection, blood samples were taken for serum cytokine immediately. Fifty healthy children from outpatient clinics were randomly selected as the normal control while assessing the cytokine levels.

### Ethics statement

This work was approved by the ethics committee of the Children’s Hospital, Zhejiang University School of Medicine, China. The patient records/information were anonymized and de-identified prior to analysis.

### Antimicrobial susceptibility testing

Identification and antimicrobial susceptibility testing of ABC were performed with the Vitek2 Compact automated system, using annually published Clinical and Laboratory Standards Institute breakpoints [16] (tigecycline, using Food and Drug Administration breakpoints [17]). Non-MDR ABC isolates were defined as resistant to two or fewer drug classes. MDR ABC was defined as isolates resistant to more than or equal to three drug classes [18].

### Cytokine

Blood samples were collected using a separation gel vacuum tube and centrifuged at 1,000 g at 20°C for 20 min after clotting. The serum was carefully harvested and subjected immediately to Th1/Th2 cytokine measurement using flow cytometry. Then, a cytometric bead array (CBA) kit (BDTM CBA human Th1/Th2 cytokine kit II; BD Biosciences, CA, USA) was used to determine quantitatively the concentration of cytokines. The CBA technique was based on six bead populations with distinct fluorescence intensities, which had been coated with specific capture antibodies. The fluorescent dye had an emission wavelength maximum of 650 nm (FL-3), which was detectable using flow cytometry. The cytokine capture beads were mixed with the phycoerythrin-conjugated detection antibodies and then incubated with recombinant standards or test samples to form sandwich complexes. Following the acquisition of sample data on a FACSCalibur flow cytometer (Becton Dickinson, CA, USA), the sample results were displayed in graphical and tabular formats using the BD CBA Software (BD Biosciences, CA, USA). A standard curve was set up for each individual set of reagents. The minimum and maximum limits of detection for all cytokines were 1.0 and 5,000 pg/mL, respectively.

### Statistical analysis

The WHONET software was used to analyze the antibiotic susceptibility of ABC [19]. Differences between categorical variables were analyzed using the chi-square test and two-tailed Fisher’s exact test, as appropriate. Univariate analysis was used to identify significant factors for MDR acquisition. Independent covariates with a *P* value less than 0.10 were included in a multivariate logistic regression analysis, with results presented as odds ratio (OR) and 95% confidence interval (95% CI). The concentrations of cytokines were compared using the Kruskal–Wallis test. Statistical significance was defined as *P* < 0.05, and all tests of significance were two-sided. SPSS 19.0 (SPSS, IL, USA) was used for all statistical analyses.

## Results

### Antibiotic susceptibility of nosocomial ABC

**Fig 1:**
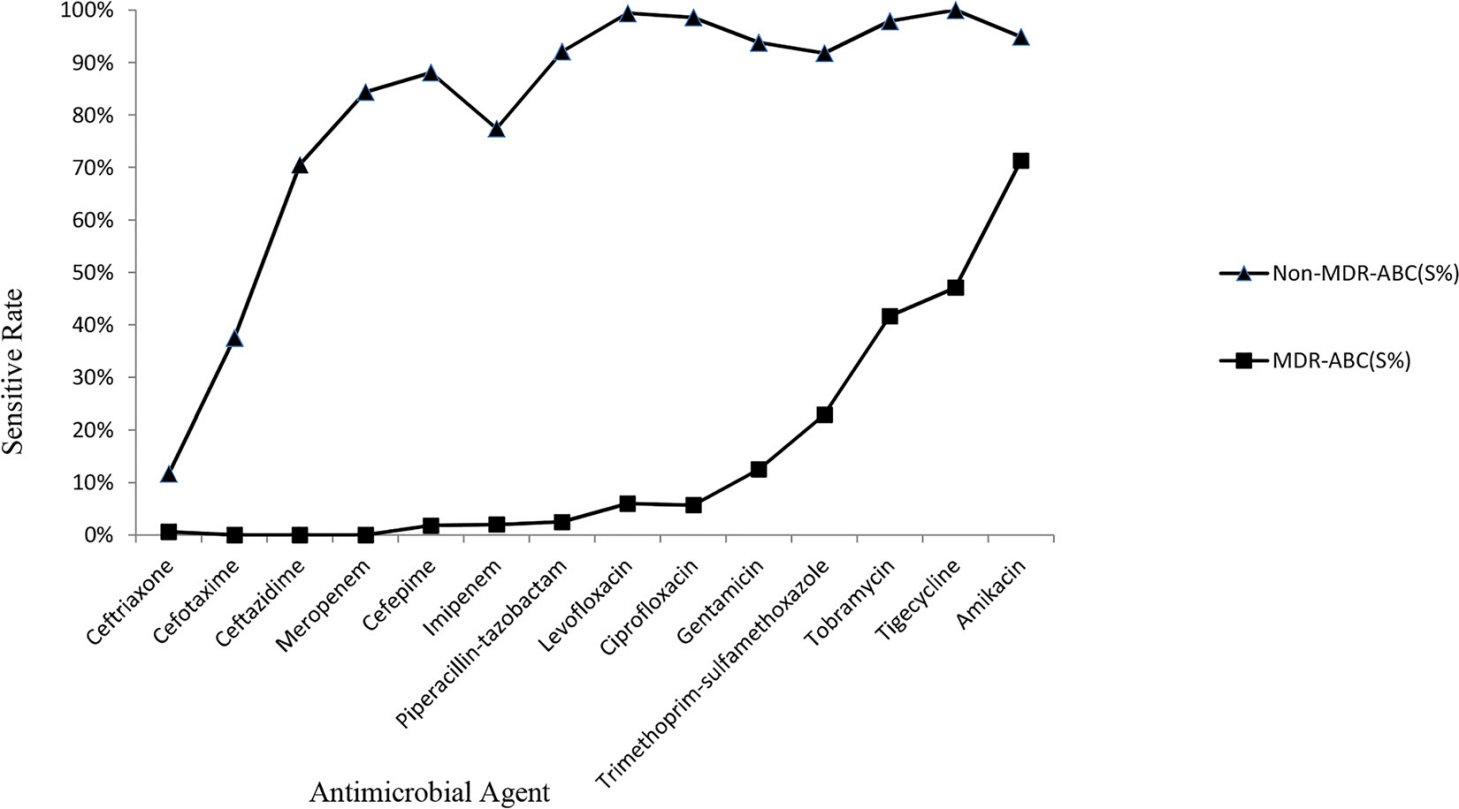
The antibiotic susceptibility of nosocomial *A*. *baumannii* complex.

After removing duplicates and excluding readmissions, 377 positive nosocomial ABC cultures were identified. According to the antibiotic susceptibility of nosocomial ABC, 200 (53.1%) nosocomial ABC isolates were defined as MDR ABC. At the same time, 177 (46.9%) nosocomial ABC isolates were defined as non-MDR ABC. The antibiotic susceptibility of nosocomial ABC is shown in Fig 1. Both non-MDR ABC and MDR ABC were highly resistant to ceftriaxone and cefotaxime. It is worth noting that MDR ABC was highly resistant to all antibiotics except amikacin (71.3%), tigecycline (47.1%), and tobramycin (41.7%). Non-MDR ABC was highly sensitive to meropenem, cefepime, imipenem, piperacillin–tazobactam, levofloxacin, ciprofloxacin, gentamicin, trimethoprim–sulfamethoxazole, tobramycin, tigecycline, and amikacin (>80%).

### Characteristics of nosocomial ABC

**Fig 2:**
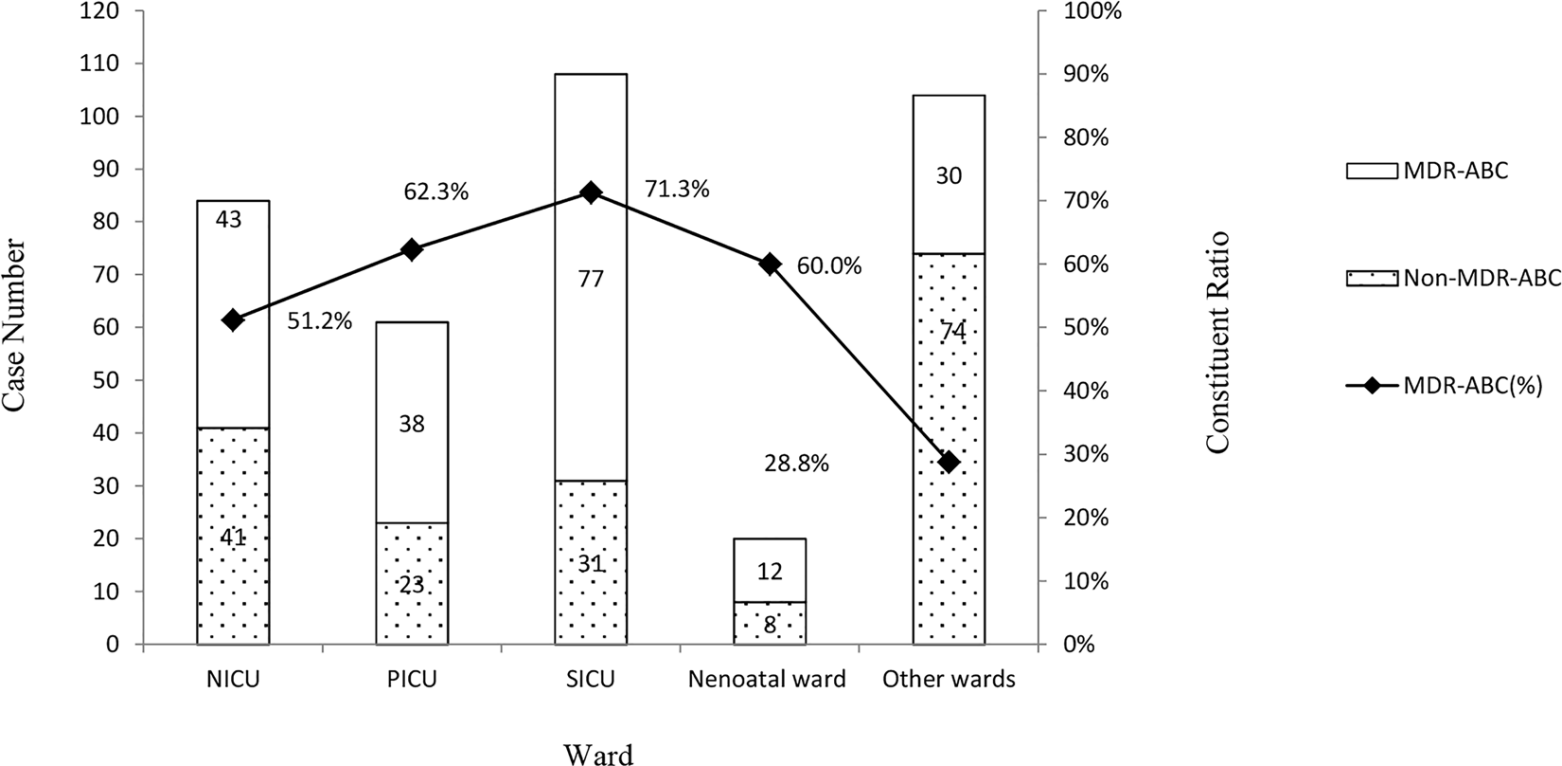
The distribution of nosocomial *A*. *baumannii* complex isolates from children in different hospital departments.

**Fig 3:**
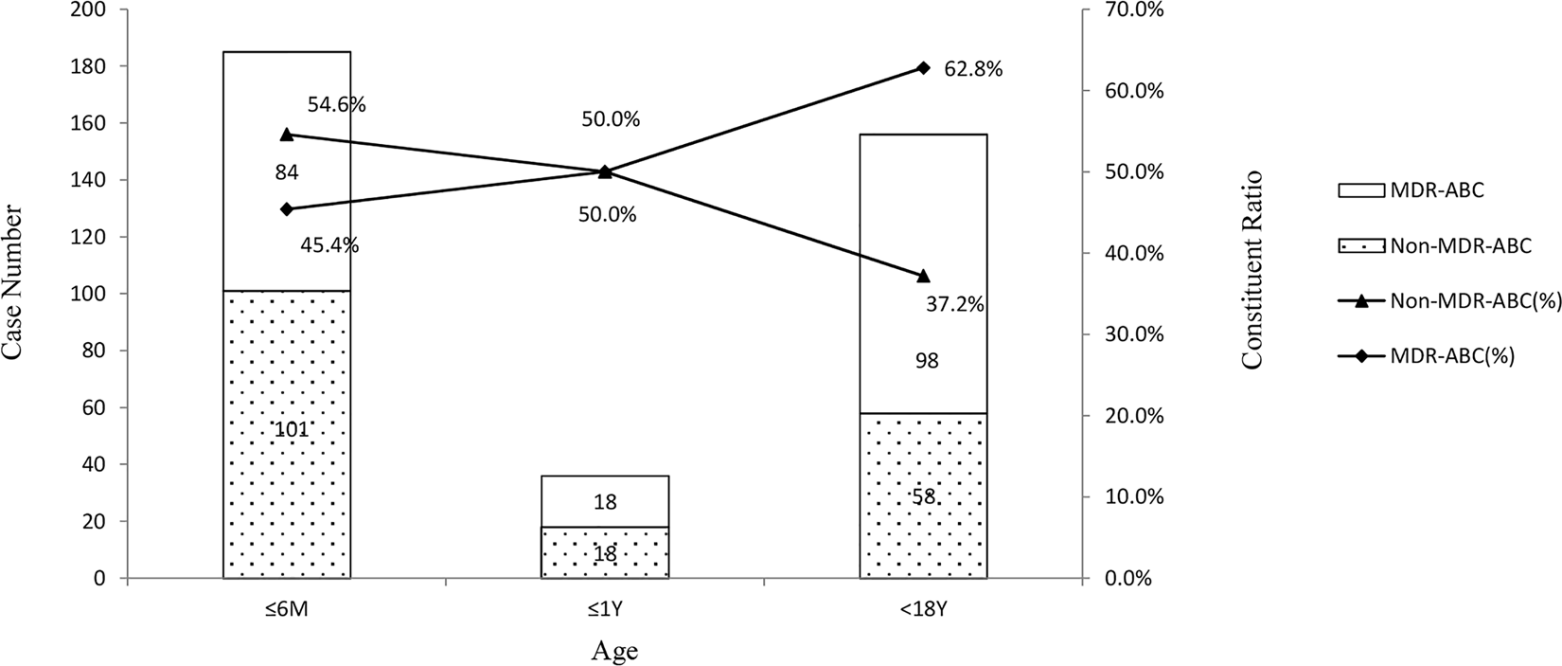
The age distribution of nosocomial *A*. *baumannii* complex isolates from children younger than 18 years.

Overall, 377 nosocomial ABC isolates were detected from 13,699 bacteria between January 1, 2011, and December 31, 2014, in a tertiary hospital in eastern China after removing duplicates. Moreover, 321 of the 377 nosocomial ABC isolates were collected from the respiratory tract and 253 from neonatal intensive care unit (NICU), pediatric intensive care unit (PICU), and surgical intensive care unit (SICU). According to the antibiotic susceptibility, 200 nosocomial MDR ABC isolates were assessed. Also, 167 of the 200 nosocomial MDR ABC isolates were collected from the respiratory tract. However, constituent ratios (52.0%) of nosocomial MDR ABC isolated from the respiratory tract were not higher than those of others (58.9%, *P* = 0.339). Also, 158 of the 200 nosocomial MDR ABC isolates were collected from the ICUs. The constituent ratio for nosocomial MDR ABC of ICUs was significantly higher than those of other wards (NICU vs other wards, *P* = 0.002; PICU vs other wards, *P* = 0; SICU vs other wards, *P* = 0). Seventy-seven of the 200 nosocomial MDR ABC isolates were collected from SICU. Among all wards, SICU had the highest constituent ratio for MDR ABC (71.3%), which was significantly higher than those for NICU (*P* = 0.004) and other wards (*P* = 0). Although 104 nosocomial ABC isolates were collected from other wards, the constituent ratio for MDR was only 28.8%. The distribution of nosocomial ABC isolates from children in different hospital departments is shown in Fig 2. The age distribution of nosocomial ABC isolates from children younger than 18 years is shown in Fig 3. The constituent ratios for nosocomial MDR ABC were 45.4%, 50%, and 62.8% in children aged 0–6 months, 6 months to 1 year, and 1–18 years, respectively. The constituent ratio for nosocomial MDR ABC among children aged more than 1 year was significantly higher than those for nosocomial MDR ABC among children aged 0–6 months (*P* = 0.001). Additionally, the 30-day mortality of children with nosocomial MDR ABC (31.0%) was significantly higher than the 30-day mortality of children with nosocomial non-MDR ABC (0.73%, *P* = 0).

### Risk factors for acquisition of nosocomial MDR ABC

The demographic and clinical characteristics of children in MDR and non-MDR groups are presented in Table 1. On univariate analysis, the following were found to be associated with the acquisition of nosocomial MDR ABC: older than 1 year (*P* = 0.001), gender (*P* = 0.06), being in the ICUs (*P* = 0), being in the SICU (*P* = 0), congenital heart disease (*P* = 0.078), prolonged hospital stay (*P* = 0), surgical intervention (*P* = 0), and mechanical ventilation (*P* = 0). On multivariate logistic analysis, being in the SICU (OR, 2.735; 95% CI, 1.273–5.873; *P* = 0.01), prolonged hospital stay (OR, 1.008; 95% CI, 1.000–1.015; *P* = 0.038), surgical intervention (OR, 2.876; 95% CI, 1.733–4.775; *P* = 0), and mechanical ventilation (OR, 2.545; 95% CI, 1.522–4.257; *P* = 0) were found to be independent risk factors for MDR acquisition among children with nosocomial ABC.

**Table 1:** Comparison of risk factors among children with nosocomial MDR *A*. *baumannii* complex and nosocomial non-MDR *A*. *baumannii* complex: univariate analysis.

Variables	MDR-ABC (*n* = 200)	Non-MDR-ABC (*n* = 177)	*P*-value
Age (>1 year)	98 (49.0)	58 (32.8)	**0.001**
Male	118 (59.0)	121 (68.4)	0.06
Patient location			
ICU	158 (79.0)	95 (53.6)	**0**
SICU	77 (38.5)	31 (17.5)	**0**
Reason for admission			
Congenital heart disease	89 (44.5)	63 (35.6)	0.078
Pneumonia	122 (61.0)	120 (67.8)	0.17
Premature delivery	29 (14.5)	30 (16.9)	0.514
Tumors	8 (4)	6 (3.4)	0.755
Median of hospital stay(day)	34	20	**0**
Previous hospitalization	33 (16.5)	37 (20.9)	0.272
Surgical intervention	122 (61.0)	65 (36.7)	**0**
Mechanical ventilation	108 (54.0)	54 (30.5)	**0**

Non-MDR-ABC: non-multidrug-resistant *A*. *baumannii* complex

MDR-ABC: multidrug-resistant *A*. *baumannii* complex

ICU: intensive care unit

SICU: surgical intensive care unit.

### Cytokine levels of children with nosocomial ABC

**Fig 4:**
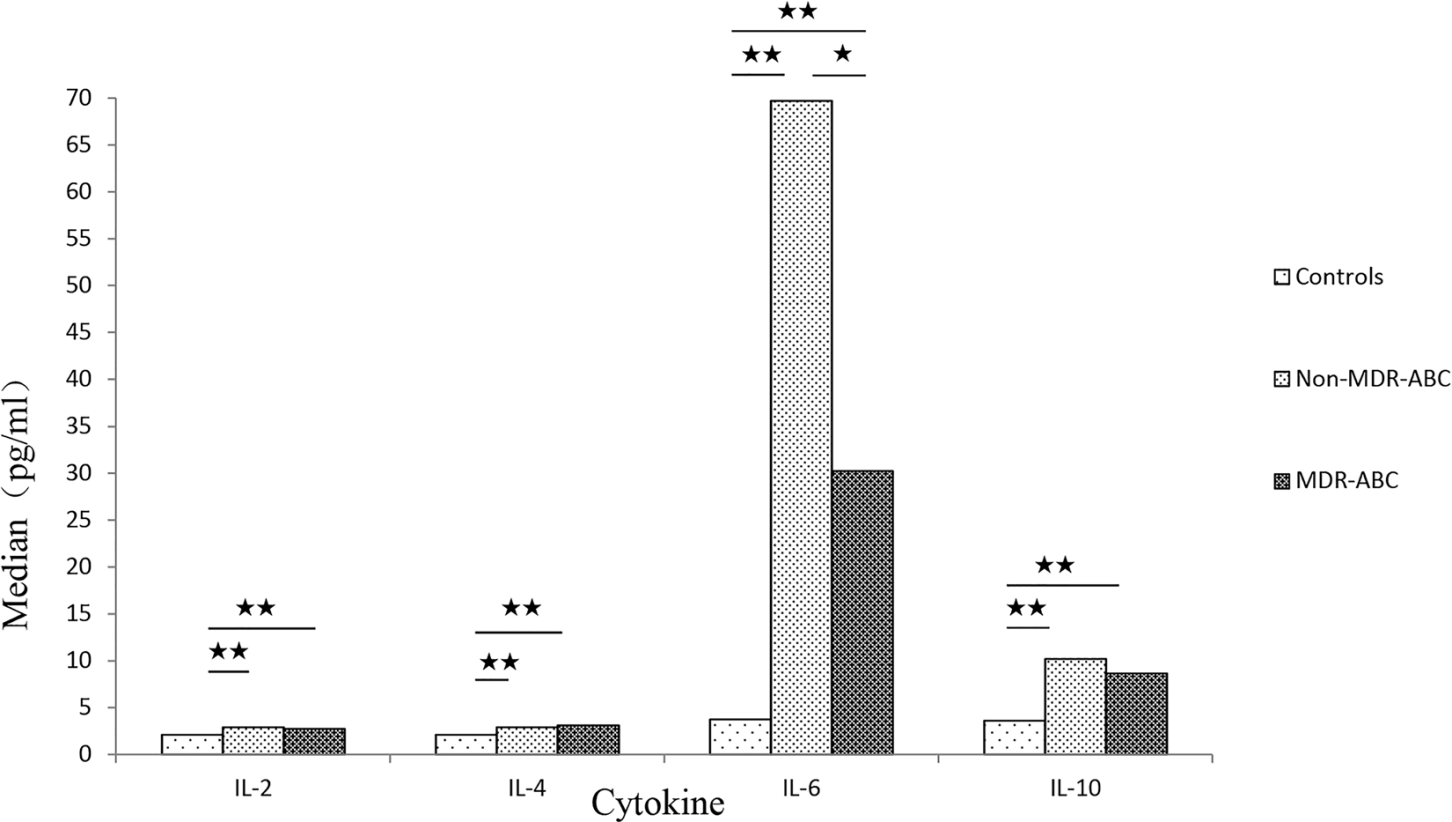
IL-2, IL-4, IL-6, and IL-10 levels of children with nosocomial *A*. *baumannii* complex.

The levels of IL-2, IL-4, IL-6, and IL-10 in children with nosocomial non-MDR ABC or nosocomial MDR ABC were significantly higher than those in the control group without nosocomial non-MDR ABC and nosocomial MDR ABC. The IL-6 level of children with nosocomial MDR ABC was significantly lower than that of the children with nosocomial non-MDR ABC. However, no significant variation in IL-2, IL-4, and IL-10 levels was found between nosocomial non-MDR ABC and nosocomial MDR ABC. The results of the comparison are shown in Fig 4.

## Discussion

As one of the most important opportunistic pathogens, nosocomial ABC causes serious health care–associated complications in critically ill patients [20]. The rise in nosocomial MDR ABC infections has been a serious concern worldwide with the increasingly widespread use of broad-spectrum antibiotics. Although many studies across the world on nosocomial MDR ABC have been reported, they were mostly focused on adults [21–23]. This study was executed because the data focusing on children with nosocomial MDR ABC were lacking.

In total, 377 positive nosocomial ABC cultures were identified, and 53.1% nosocomial ABC isolates were defined as MDR ABC. Nosocomial MDR ABC is highly prevalent in a 1000-bed tertiary hospital in eastern China, and hence the treatment of this infection has become a big challenge. According to the antibiotic susceptibility, both nosocomial non-MDR ABC and nosocomial MDR ABC were found to be highly resistant to ceftriaxone and cefotaxime. Thus, these drugs are not recommended in clinical treatment. Nosocomial MDR ABC was highly resistant to almost all antibiotic families. It has already seriously restricted the therapeutic options for nosocomial MDR ABC infection. However, nosocomial MDR ABC was sensitive to amikacin (71.3%), tigecycline (47.1%), and tobramycin (41.7%), which may be effective in the treatment process. Additionally, the combined antibiotic therapy may be the second effective strategy for treating nosocomial MDR ABC infection [24, 25].

Nosocomial ABC in children mainly originates from the respiratory tract and ICUs. Nosocomial MDR ABC also originates from the respiratory tract, but the constituent ratios (52.0%) of MDR ABC isolated from the respiratory tract were not higher than those of others (58.9%, *P* = 0.339). The distribution of MDR of nosocomial ABC in specimens is not significantly different. The distribution in the hospital departments indicates that nosocomial ABC mainly originates from the ICUs and the constituent ratio for nosocomial MDR ABC of the ICUs is significantly higher than those of other wards (NICU vs other wards, *P* = 0.002; PICU vs other wards, *P* = 0; SICU vs other wards, *P* = 0). It is consistent with the conclusion of other studies focused on adults. This study further analyzed the cases from the ICUs and found that SICU has the highest constituent ratio for nosocomial MDR ABC (71.3%), which is significantly higher than those of NICU (*P* = 0.004) and other wards (*P* = 0). This conclusion was not arrived at in the other reports. The age distribution showed that the constituent ratios for nosocomial MDR ABC exhibited a significant increase among children older than 1 year (62.8%). Therefore, the relevant monitoring and protection associated with nosocomial ABC infection should be strengthened in older children. Although the 30-day mortality of children with nosocomial MDR ABC (31.0%) was significantly higher than that of children with nosocomial non-MDR ABC (0.73%, *P* = 0), these rates were lower than those of adults [1].

According to previous reports, the risk factors for patients with MDR ABC were associated independently with prolonged hospital stay, previous hospitalization, mechanical ventilation, surgical intervention, and various reasons for admission [1, 8–12]. In this study, being in SICU (OR, 2.735; 95%CI, 1.273–5.873; *P* = 0.01), prolonged hospital stay (OR, 1.008; 95% CI, 1.000–1.015; *P* = 0.038), surgical intervention (OR, 2.876; 95% CI, 1.733–4.775; *P* = 0), and mechanical ventilation (OR, 2.545; 95% CI, 1.522–4.257; *P* = 0) were independent risk factors for MDR acquisition among children with nosocomial ABC. It was not mentioned in the other studies that being in the SICU was an independent risk factor for MDR acquisition among children with nosocomial ABC. The present results indicate the importance of strengthening the supervision of nosocomial MDR ABC infection in the SICU. The aforementioned conclusion may not be applicable to other hospitals because the cases included in the present study were all children from a tertiary hospital in eastern China; they were different from other studies in terms of geographical distribution, age, etiology, and treatment options. The aforementioned risk factors were determined only under the conditions of the present study.

Using the Th1/Th2 kit, the cytokine levels of children with nosocomial ABC were assessed. No significant change was found in other cytokines (excluding IL-2, IL-4, IL-6, and IL-10). So, only the IL-2, IL-4, IL-6, and IL-10 levels of children with nosocomial ABC are presented in this study. Patients with cancer and autoimmune disease, which would interfere with the cytokine levels, were excluded [26–30]. IL-2, IL-4, IL-6, and IL-10 levels of children with nosocomial non-MDR ABC or nosocomial MDR ABC were significantly higher than those of the control group. The present results indicate that IL-6 is worthy of attention in children infected with nosocomial MDR ABC. The present study found the IL-6 level of children with nosocomial MDR ABC to be significantly lower than that of children with nosocomial non-MDR ABC. However, similar findings were not reported for IL-2, IL-4, and IL-10. IL-6 is an important soluble mediator with a pleiotropic effect on immune response, inflammation, and hematopoiesis. For example, as the strongest activator of the hepatic acute-phase response, IL-6 mediates the secretion of a group of proteins, including C-reactive protein, serum amyloid A, haptoglobin, and α_1_-acid glycoprotein during bacterial infections [31–33]. It is suspected that IL-6 may be involved in some kind of immune regulation mechanism in children with nosocomial MDR ABC infection, which needs further investigation. It may be of great significance in treating nosocomial MDR ABC infection.

In conclusion, this study showed that nosocomial MDR ABC infection is a serious concern in pediatric patients. Being in the SICU, prolonged hospital stay, surgical intervention, and mechanical ventilation increased the risk of developing nosocomial MDR ABC infection in children. IL-6 might play an important role in immune regulation in children with nosocomial MDR ABC infection.

## Supporting Information

S1 Data Date set. (XLSX)
